# Complete elliptical ring geometry provides energy and instrument calibration for synchrotron-based two-dimensional X-ray diffraction

**DOI:** 10.1107/S0021889813022437

**Published:** 2013-09-18

**Authors:** Michael L. Hart, Michael Drakopoulos, Christina Reinhard, Thomas Connolley

**Affiliations:** a Diamond Light Source Ltd, Diamond House, Harwell Science and Innovation Campus, Didcot, Oxfordshire OX11 0DE, UK

**Keywords:** instrument calibration, synchrotron radiation, X-ray diffraction

## Abstract

A complete calibration method to characterize a static planar two-dimensional detector for use in X-ray diffraction at an arbitrary wavelength is described. This method is based upon geometry describing the point of intersection between a cone’s axis and its elliptical conic section. This point of intersection is neither the ellipse centre nor one of the ellipse focal points, but some other point which lies in between. The presented solution is closed form, algebraic and non-iterative in its application, and gives values for the X-ray beam energy, the sample-to-detector distance, the location of the beam centre on the detector surface and the detector tilt relative to the incident beam. Previous techniques have tended to require prior knowledge of either the X-ray beam energy or the sample-to-detector distance, whilst other techniques have been iterative. The new calibration procedure is performed by collecting diffraction data, in the form of diffraction rings from a powder standard, at known displacements of the detector along the beam path.

## Abstract 2

A complete method for calibrating a two-dimensional flat-panel detector for use in X-ray diffraction is described. The method provides both the beam energy and the sample-to-detector distance. The geometry for the intersection of a cone’s axis and its elliptical conic section is also presented.

## 1. Introduction

The use of two-dimensional detectors is widespread in the collection of X-ray diffraction data. Their high resolution and suitability for use at high X-ray energies allows a variety of experiments to be performed, such as protein crystallography (Helliwell, 1982 ▶), single-crystal diffraction (Riekel, 2000 ▶), the study of grain orientation and strain in polycrystals (Hofmann *et al.*, 2012 ▶), the evolution of twinning in response to strain (Evans *et al.*, 2012 ▶), and the study of thick samples in complex environments (Sui *et al.*, 2010 ▶).

We present a non-iterative method for the calibration of a planar two-dimensional detector used for synchrotron-based X-ray diffraction at an arbitrary wavelength. The calibrated parameters include the sample-to-detector distance, the position of the X-ray beam trajectory on the detector, the detector tilt and the beam energy. Our approach is made possible through the presentation of a single coherent geometrical description and uses diffraction ring data collected from a number of detector positions along the beam path. Our method accounts for the random variation in detector tilt and lateral position as the detector is moved during data collection. This calibration technique is useful in a facility where the energy can be freely chosen but difficult to evaluate accurately *via* other methods.

There are many diffraction geometries in use for the collection of X-ray crystallographic data, and these have previously been described (Helliwell, 2004 ▶). Here we are concerned with the geometry of the detector relative to the X-ray beam. Where possible, we have adopted a notation used elsewhere (Hinrichsen *et al.*, 2008 ▶). A description for the geometry of the intersection of a cone’s axis and its elliptical conic section has been attempted elsewhere (Hinrichsen *et al.*, 2008 ▶) and will be discussed in more detail later. We do not consider parabolas or hyperbolas, and thus our method applies to cases where both the energy is high enough and the detector’s tilt is set appropriately such that calibration data from a powder standard can be recorded as ellipses upon the detector surface.

At low energies, the diffraction pattern of a calibration standard can be recorded and the topology of that pattern used to estimate the diffraction geometry and incident wavelength (Norby, 1997 ▶). This method can be sufficiently accurate at large X-ray wavelengths (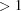
 Å), when large diffraction angles are present. With two-dimensional detectors, this indirect calibration method is common practice, owing to its practical implementation in various software packages (Heiney, 2005 ▶; Hammersley *et al.*, 1996 ▶; Ilavsky, 2012 ▶) which are currently used in many facilities around the world.

With a two-dimensional detector it is possible and often desirable to conduct the diffraction experiment at short wavelengths – or high photon energies – in order to collect a large section of reciprocal space. At high X-ray energies, the small diffraction angles (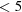
°) lead to an interaction between the sample-to-detector distance and the wavelength that can be hard to separate mathematically when solving the relevant nonlinear equations (Bragg’s law). This interaction makes their simultaneous refinement difficult (Hong *et al.*, 2012 ▶).

A diffraction ring resulting from an interplanar spacing given by *d*, recorded on an orthogonal detector at a distance of *D* from the sample, using high-energy X-rays of wavelength λ, will have a radius approximately given by 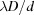. It is not possible to solve this equation for both λ and *D* using diffraction data taken from a single sample-to-detector distance. At best the product of λ and *D* can be found. Thus, at high energies, it is often the case that either the sample-to-detector distance or the beam wavelength needs to be accurately determined prior to the refinement of the other calibration parameters. One possible method to indirectly determine the incident wavelength is to configure the monochromator to produce a wavelength that matches the absorption edge of a known sample with a well defined *K* edge; however, this will only cover a discrete set of energies and not always the desired one. An alternative method for calibration at high energies, which uses the extra information obtained by varying the position of the detector, exists (Hong *et al.*, 2012 ▶) but is iterative in its application and thus requires the design of an exit strategy.

**Figure 1:**
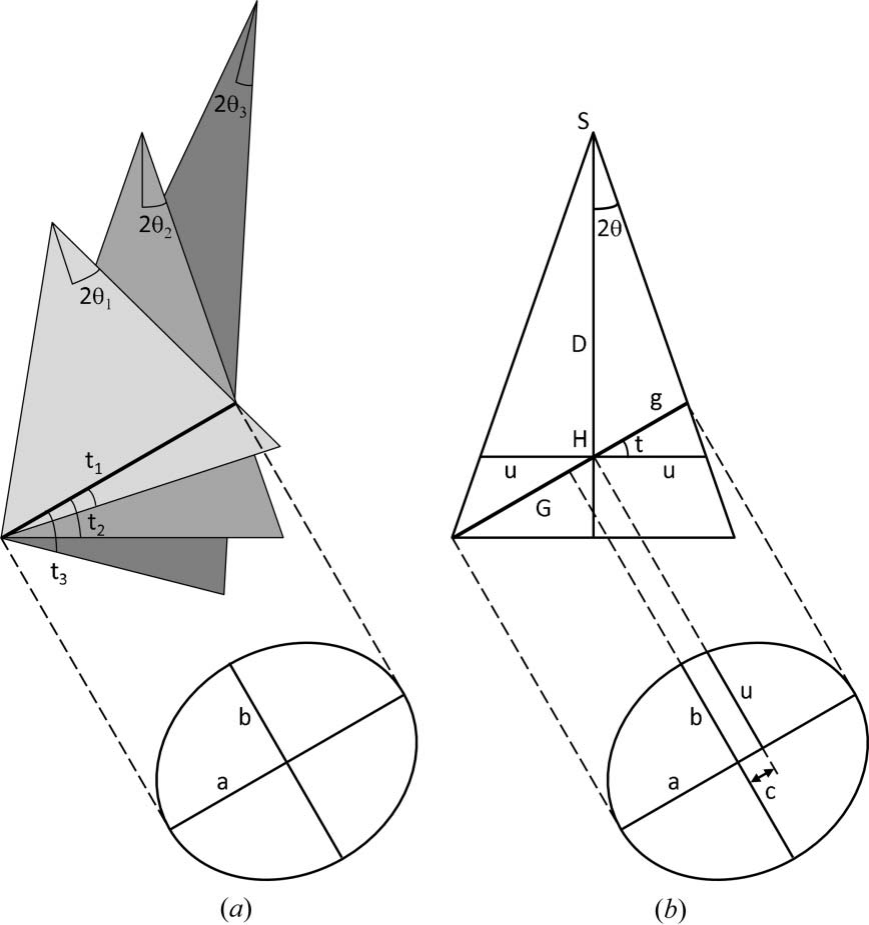
Geometry of a plane intersecting a cone, representing a two-dimensional detector intersecting a diffraction cone. (*a*) A single elliptical conic section cannot define the cone from which it came. (*b*) Plan view showing the geometry of a plane (thick line) intersecting a cone of opening angle 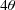, where θ is equivalent to Bragg’s angle in the context of a powder sample diffracting to produce a diffraction cone. The projection of the resulting elliptical conic section is shown. (Diagrams are not drawn to scale.)

Another difficulty with calibrating a two-dimensional detector using diffraction ring data is that determination of the detector tilt relative to the beam direction is nontrivial. The eccentricity of a diffraction ring,^1^ seen as an ellipse, can be expressed as a function of the detector tilt and the Bragg angle [see equation (11) derived later]; as such it is impossible to calculate the detector tilt using a single diffraction ring without knowledge of the Bragg angle (which in itself is a function of the interplanar spacing and the beam wavelength). Fig. 1 ▶(*a*) demonstrates this ambiguity, where it can be seen that cones of various opening angle and orientation can intersect a plane to produce an ellipse of identical eccentricity.

For each diffraction cone, as seen in Fig. 1 ▶(*a*), that projects to give an identically shaped diffraction ring, the location of the beam centre within each ellipse will differ as per equation (5). The beam centre is not located at either the ellipse centre or the ellipse focal point of a diffraction ring [see equation (10) derived later]. As a consequence the beam centre cannot be defined from one diffraction ring. For a set of concentric cones the positions of the ellipse centres and the ellipse focal points differ for rings originating from different (*hkl*) planes. This viewpoint is in contrast to that presented elsewhere (Norby, 1997 ▶; Rajiv *et al.*, 2007 ▶; Hinrichsen *et al.*, 2008 ▶), where it was incorrectly stated that the cone’s axis intersects the elliptical conic section at its focal point. Subsequent geometrical expressions involving the focal point were thus incorrectly derived (Rajiv *et al.*, 2007 ▶; Hinrichsen *et al.*, 2008 ▶).

By using more than one diffraction ring it is possible to derive values for the detector tilt and the X-ray energy using equation (11), if the interplanar spacings for each diffraction ring are known. However, for small Bragg angles or detector tilt 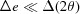. Equation (11) becomes insensitive to the precise value of the Bragg angle and so cannot be used to attain a good value for the beam energy. To overcome this problem, we use diffraction ring data collected from multiple detector distances to first calculate the beam energy and then give a value for the detector tilt.

By using data from multiple detector positions of known separation we can solve for both λ and *D*. Our method considers geometries where the detector normal may exhibit an arbitrary angle relative to the incoming beam, regardless of whether the tilt is applied intentionally or not. It is important to allow for a variation in tilt, due to mechanical runout, as the detector stage is translated whilst collecting diffraction data for calibration. For this reason simple ray tracing techniques may be inaccurate.

The closed form solution we present here yields results for all calibration parameters in a non-iterative manner and at the same time adds understanding to the underlying geometrical problem. Iterative techniques (Hong *et al.*, 2012 ▶) lacking a complete model may yield sufficiently accurate results, if the exit strategy is suitably designed.

**Figure 2:**
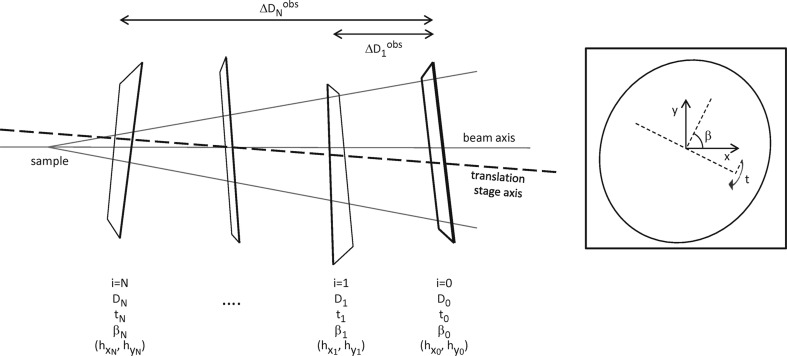
The calibration procedure permits both the detector tilt and the lateral position of the detector to change in both a systematic and unknown manner, as the detector is translated along the beam path. For the detector tilt we use a convention of a tilt angle, *t*, which occurs in a direction denoted by β.

The quality of our method derives from a complete analytical three-dimensional model which utilizes powder diffraction patterns from a standard, recorded at different sample-to-detector distances (Fig. 2 ▶). This allows us to remove the ambiguity in sample-to-detector distance and wavelength (Bragg’s law) as well as the ambiguity in detector tilt and opening angle of a diffraction cone (Fig. 1 ▶ *a*).

**Figure 3:**
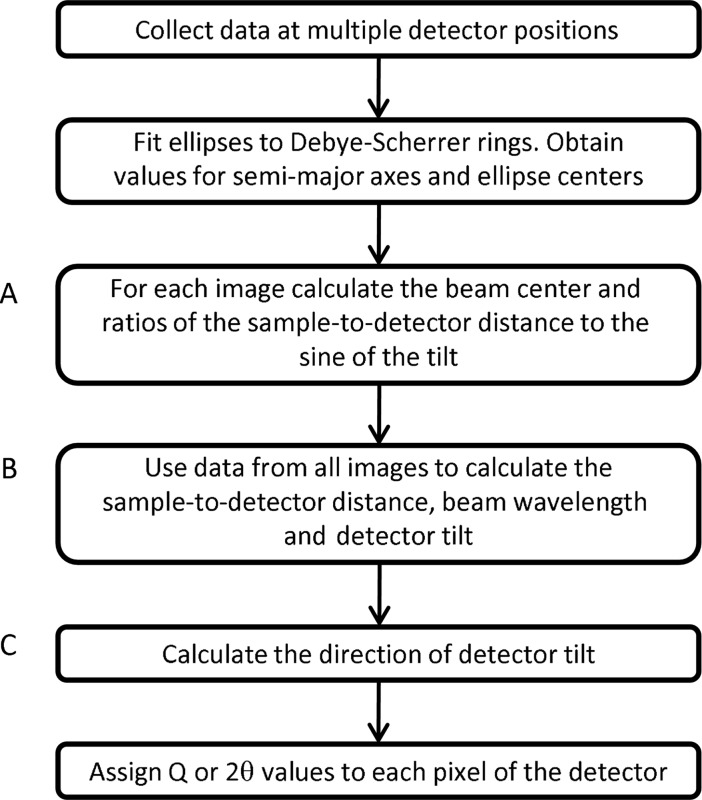
Flow chart showing the steps required to calibrate a two-dimensional detector. Steps labelled A, B and C indicate the part of the procedure from which calibration parameters are determined.

Our method can be broken down into a number of distinct steps (Fig. 3 ▶). Each diffraction ring recorded on a two-dimensional detector is an ellipse. The location of the ellipse centre and the length of the semi-major axis in the coordinate system of the detector need to be accurately determined. Diffraction data are collected from a number of different nominal sample-to-detector distances. At each detector position, the ratio of the sample-to-detector distance to the sine of the tilt and the location of the beam centre on the detector are found by solving a nonlinear equation. Data from all detector positions are then brought together in another nonlinear equation which provides values for the sample-to-detector distance and the beam wavelength. Finally, the detector tilt and direction of tilt can be calculated from the sample-to-detector distance and the beam centre, respectively. By using data from all available diffraction rings we can improve the accuracy of these calculated parameters. Solving an overdetermined system reduces the sensitivity of the calculated parameters to noise.

Having fully calibrated the detector in terms of its five spatial parameters and the incident beam wavelength, reciprocal space values, *Q*, can be assigned to each detector pixel. This can be achieved by using equations (19) and (20).

The method presented in this article requires the parameterization of the elliptical diffraction ring formed at the intersection of the diffraction cone and the plane of the two-dimensional detector. This procedure is beyond the scope of this article; however, a number of techniques are known (Hanan *et al.*, 2004 ▶; Hart & Drakopoulos, 2013 ▶). The technique used in this article directly fits an intensity-weighted ellipse to peak centres determined at equal intervals in azimuthal angle. This technique works well for both complete and spotty rings (Hart & Drakopoulos, 2013 ▶).

To aid our attempt in providing an accurate method for detector calibration, we first present a complete geometrical description of the intersection between a cone’s axis and its elliptical conic section.

## 2. Ellipse geometry – the intersection of a cone’s axis and its elliptical conic section

If a two-dimensional detector is positioned perfectly orthogonal to the X-ray beam, then a diffraction ring will appear as a circle. A deviation from orthogonality will result in an elliptical ring. The right-hand diagram of Fig. 2 ▶ demonstrates the convention used in this article in the case where a detector exhibits a tilt. In the plane of an orthogonal detector we consider a single tilt, *t*, of the detector, whilst the azimuthal direction of the tilt relative to the *x* axis, β, is perpendicular to the axis of tilt. We use a subset of the Euler angles.

Fig. 1 ▶(*b*) shows a view (Kumar, 2006 ▶) along the tilt axis of the detector, depicting the X-ray beam (propagating along line S–H), a diffraction cone of half-angle 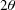, where θ is the Bragg angle, and a representation of the detector surface (bold off-horizontal line) intercepting the cone. The intersection of a cone and the two-dimensional detector (placed at an angle *t*) is an ellipse. This ellipse represents the recorded diffraction ring on the detector. The ellipse is shown projected from the detector surface, and both the semi-major, *a*, and semi-minor, *b*, axes are indicated. The distance from the centre of the ellipse to the point where the X-ray beam strikes the detector surface, *c*, is also indicated. *D* is the distance from the sample, *S*, to the detector at *H*. Labelled on both the plan view and the ellipse, *u* is the radius of a ring as it would be recorded on an orthogonal, 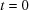, detector at position *H*.

Other authors have stated that the direct X-ray beam or axis of a cone intersects a detector or plane at the focal point of the diffraction ring or ellipse (Norby, 1997 ▶; Rajiv *et al.*, 2007 ▶; Hinrichsen *et al.*, 2008 ▶) or at a symmetry centre defined by the diffraction ellipses (Cervellino *et al.*, 2006 ▶, 2008 ▶). We postulate that, should a cone intersect a plane to produce an elliptical conic section, then the cone axis intersects the plane at a point that lies between the ellipse centre and closest focal point of the ellipse. This can be visualized by considering the three-dimensional geometrical construct known as Dandelin spheres (De Villiers, 2007 ▶; Dandelin, 1822 ▶).

A number of expressions describing the intersection of a plane and a cone, in the context of two-dimensional X-ray diffraction, can now be derived.

The similarity of the triangle spanned by *u* and *D*, the triangle spanned by 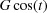 and 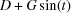 (Kumar, 2006 ▶), and the triangle spanned by 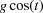 and 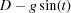 gives the following geometrical relationship:
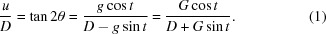



Using the substitutions 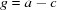 and 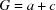, equation (1) can be rewritten to give the semi-major axis, *a*, in terms of the sample-to-detector distance, the detector tilt and the distance from the ellipse centre to the beam centre:
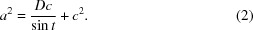



Expressions for *a* and *c* can also be found in terms of the sample-to-detector distance, the detector tilt and the Bragg angle. Equation (1) gives
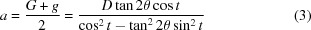
and
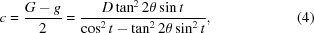
and thus
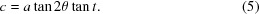
Equations (3) and (4) are equivalent to expressions derived elsewhere (Hinrichsen *et al.*, 2008 ▶).

In polar coordinates, the distance from the ellipse centre to any point on the ellipse as a function of the angular coordinate, γ, measured from the major axis is
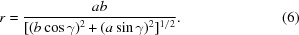



Use of the substitutions 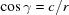 and 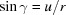 gives an expression for the semi-minor axis, *b*, in terms of the sample-to-detector distance, the detector tilt and the Bragg angle:
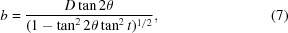
and thus
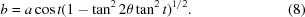



The distance from the centre of the ellipse to the focus, *f*, can now be given:
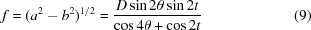
and
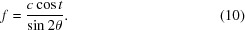



The eccentricity of the ellipse, *e*, can also be described:
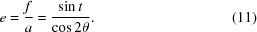



Our expressions for the semi-minor axis and the eccentricity [equations (7) and (11), respectively] disagree with those derived elsewhere (Hinrichsen *et al.*, 2008 ▶). We do, however, agree with Hinrichsen in that the eccentricity is a function of the tilt, *t*, and the Bragg angle, θ, which is in contrast to an alternative view that a single value for eccentricity can be used to describe the detector tilt (Cervellino *et al.*, 2006 ▶, 2008 ▶). Furthermore, it is possible to conclude from equation (10) that, if the detector tilt is such that the detector intersects the diffraction cone to form an ellipse, then the point where the cone’s axis intercepts the ellipse is closer than either focal point is to the ellipse centre. In other words, if 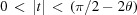, then 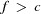.

Directly derived from the Cartesian equation of an ellipse, the following relationship linking the semi-major axis, *a*, the semi-minor axis, *b*, the distance from the ellipse centre to the beam centre, *c*, and the radius of the ring on an orthogonal detector, *u*, holds:
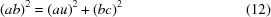



The expressions derived are summarized in Table 1 ▶.

This geometrical description provides a framework upon which our calibration procedure can be based.

## 3. Experiment and method

The calibration of the diffraction geometry for a two-dimensional flat-panel detector was carried out at the Joint Engineering, Environmental and Processing beamline (I12) at Diamond Light Source Limited (UK). The detector used was a flat-panel Pixium RF4343 (Thales), with a pixel size of 148 × 148 µm. The Pixium family of detectors is widely used for high-energy X-ray diffraction (Daniels & Drakopoulos, 2009 ▶; Sui *et al.*, 2010 ▶; Evans *et al.*, 2012 ▶; Hofmann *et al.*, 2012 ▶). Note that this article does not address any spatial distortion which may result from the curvature of a generic two-dimensional detector. Images used in our calibration procedure should already be corrected for such distortions.

A monochromatic beam with a nominal energy of 88.012 keV was generated using a double-crystal Laue X-ray monochromator. The energy was set by scanning the monochromator through the *K*-shell absorption edge of 125 µm-thick lead foil. The beam size was defined to be 200 × 200 µm, using a set of tungsten slits positioned before the sample stage.

Calibration data were collected, in transmission mode, from a fine powder cerium dioxide (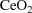) standard (NIST Standard Reference Material 674b). The powder standard was mounted in a 0.5 mm-thick planar arrangement so as to minimize any azimuthal geometrical sample effects typically produced by a sample in a capillary arrangement (Norby, 1997 ▶). The detector was fixed to a high-precision translation stage (Advanced Design Consulting USA Inc.), allowing the detector to be positioned at different sample-to-detector distances. All data were collected on the Pixium detector in high-resolution mode (2880 × 2881 pixels) with an exposure of 4.0 s.

Data were collected for two commonly occurring situations: the detector was positioned to be orthogonal (as determined by the back reflection from a guidance laser), and the detector was positioned with a nominal horizontal tilt of 6.4°. In both cases data were collected at eight nominal sample-to-detector distances, from 580 to 1980 mm in steps of 200 mm.

All data analysis was performed using custom-written software.

## 4. Beam energy determination and calibration of a two-dimensional detector

Calibration of the spatial position of the two-dimensional detector and the determination of the beam energy requires the collection of two-dimensional diffraction data from a known standard at a variety of different sample-to detector distances.

Determination of each beam centre is achieved through a purely geometrical calculation. For this part of the calibration procedure the sample only needs to produce diffraction rings that can be accurately described. However, the part of our procedure that determines the sample-to-detector distance and the beam energy uses knowledge of the sample’s lattice spacing. The measurement error in the Bragg angle as measured from a fitted ellipse is greater than that of the lattice spacing from a known standard. Use of the sample’s lattice spacing allows for a more accurate calibration procedure, whilst at the same time introducing the beam energy as an important parameter. This is why a fine powder 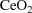 standard has been selected for use.

During data collection, the sample maintains a constant position whilst the detector is moved to different fixed positions along the beam path. It is important to know accurately the relative change in detector position along the beam path. At each detector position, diffraction data are recorded for calibration. The mechanical runout displayed by the translation stage is taken into account by our calibration procedure.

For each diffraction ring at each detector position, the ellipse centre, 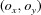, and the length of the semi-major axis, *a*, in the coordinate system of the detector are required. Also note that only equations (2) and (3) are used in our calibration procedure.

Table 2 ▶ shows a summary of the parameters that are to be calculated in order to calibrate a two-dimensional detector for use in X-ray diffraction. The required observables are also summarized. To aid understanding we segregate fitted parameters, with the superscripts fitA, fitB and fitC indicating which part of the procedure they are calculated from (Fig. 3 ▶).

### 4.1. Beam centre calculation

To calculate the beam centre, the parameters describing the diffraction ellipses from different 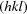 are required.

The location of the beam centre, at a given detector position, can be calculated from the data provided in a single diffraction image containing multiple rings. We calculate the location of the beam centre for multiple detector positions.

As a result of translation stage runout, the beam centre on a diffraction image is likely to be nonstationary as the detector is translated along the beam’s path. For a given image, the recorded diffraction rings result from diffraction cones with a range of opening angles 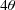. These diffraction cones share a common axis, and thus a common beam centre exists within the diffraction image for all diffraction rings. The ellipses that describe the diffraction rings do not, however, share a single common ellipse centre; instead the ellipse centres are positioned along a line coincident with the major axes of the ellipses. The beam centre is also located along this line, and the distance from the beam centre to each ellipse centre can be given by equation (4).

For each detector position, *i*, employing the method of linear least squares, the ellipse centres are used to give a line of best fit with intercept, 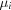, and gradient, 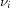. Each line of best fit can be considered to be coincident with the major axis of the ellipse, and thus to relate the *x* and *y* coordinates of the beam centre, 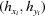:
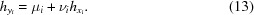



For diffraction ellipse α, where α denotes different 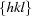, at detector position *i* we can write a general expression for the semi-major axis, 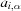. From equation (2),
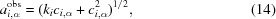
where
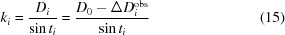
and 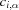 can be written as a function of the coordinates of the ellipse centre, 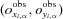, and the *x* coordinate of the beam centre, 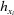, of the diffraction image:
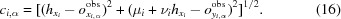


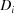
 is the sample-to-detector distance of a detector at position *i*, and 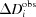 is the difference between the sample-to-detector distance at a chosen detector position, 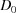, and 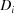. For the purpose of this calculation we have arbitrarily chosen 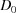 to be the distance from the sample to the detector at the detector’s furthest position from the sample as used during data collection. Calibration parameters can be found for all detector positions.

Equations (14) and (16) are used for nonlinear least-squares optimization of the *x* coordinate for the beam centre, 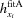, and the ratio 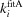 at each detector position *i*. Only data from two diffraction rings are required to solve for 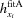 and 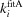; however, the use of all available rings will minimize fitting noise. The *y* coordinates of the beam centres, 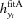, for all *i* are subsequently found using equation (13). The optimization is performed using previously determined ellipse parameters, 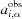 and 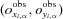 (Hart & Drakopoulos, 2013 ▶), from a number of diffraction rings that are recorded on the two-dimensional detector. For this nonlinear least-squares optimization, a good initial value for the ratio 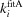 is the length of the major axis squared from the largest ellipse used, and the initial value for the *x* coordinate of the beam centre can be taken as the *x* coordinate of the ellipse centre from the smallest ellipse. The fitted value for the *x* coordinate of the beam centre is also constrained to lie to one side of all ellipse centres (Fig. 1 ▶ *b*).

**Figure 4:**
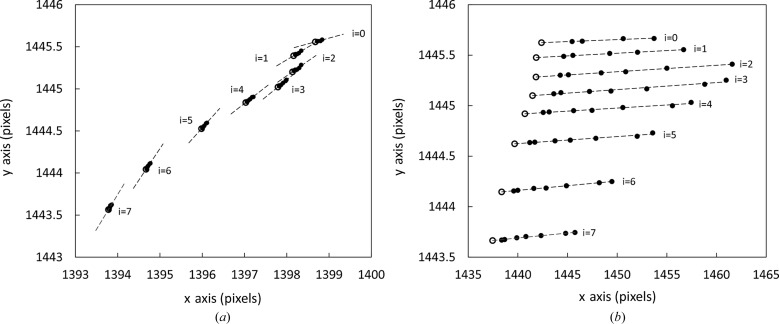
Plots showing the location of observed ellipse centres (black dots) at each detector position for the ‘orthogonal’ (*a*) and ‘tilted’ (*b*) cases. The calculated coordinates on the detector where the X-ray beam strikes the detector surface are indicated by the open black circles.

For two cases – ‘orthogonal’ (the detector is positioned orthogonal to the X-ray beam) and ‘tilted’ (the detector is positioned with a tilt measured to be approximately 6.4° about the vertical axis) – Fig. 4 ▶ shows the coordinates of the ellipse centres (dots) for eight different sample-to-detector distances, 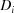. Successive images are separated by detector movements of 200.0 mm along the beam path. At each detector position, data from the (*hkl*) = (111), (002), (022), (311), (004), (422) and (333)/(511) diffraction rings were used where available, and best fit ellipses were found (Hart & Drakopoulos, 2013 ▶). For each set of ellipse centres, a line of best fit is shown together with the calculated beam centre (circles). For ideal detector displacements, the beam centres show a linear relationship in 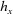 and 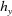. Deviations from this are caused by mechanical runout of the translation stage (Fig. 4 ▶). For the ‘tilted’ case, we can see that the ellipse centres for each image lie along straight lines; these lines are conceptually coincident with the major axes of the ellipses. In the ‘orthogonal’ case we can see that the ellipse centres also exhibit a linear relationship, which is contrary to what may be expected for an orthogonal detector (a perfectly orthogonal detector will exhibit concentric circular diffraction rings). This is due to the difficulty in aligning the detector to be perfectly orthogonal, and thus a small but quantifiable tilt remains. The small tilt coupled with the variation in the angular position of the detector results in a larger variation in the azimuthal direction of the detector tilt for the ‘orthogonal’ case as compared to the ‘tilted’ case.

### 4.2. Beam energy, sample-to-detector distance and detector tilt calculations

This part of the calibration procedure makes use of data collected from multiple detector positions. This information allows for the simultaneous determination of the beam energy and the sample-to-detector distance. Knowledge of the detector’s movement along the beam path is required.

In cases where the path of the translation stage is parallel to the beam path, the differences in distance of the detector position along the beam path, 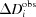, can be taken as the reported movement of the translation stage itself. If the translation stage axis is not parallel to the beam path, then the best fit values for the beam centre, 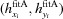, can be used to calculate 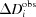. In the nonparallel case, if 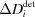 denotes the changes in reported position of the translation stage along its own axis, then 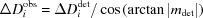, where 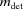 is the gradient of the best fit line relating the values 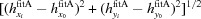 to 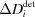.

As a function of detector position the azimuthal direction of detector tilt may be nonstationary. This does not, however, affect the accuracy of our method, as our calculation makes use of the length of the semi-major axis, *a*, and the ellipse major axes should all lie within the same plane for a given detector position.

Rewriting equation (3) as a general expression using equation (15) gives
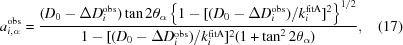
where
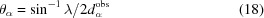
from Bragg’s law. By substituting the Bragg angles for the interplanar spacing of the crystal lattice, we not only introduce the beam wavelength as an important parameter but also improve the accuracy of our calibration procedure, as the interplanar spacings for a diffraction standard are known to a high degree of accuracy.

Equations (17) and (18) are used for nonlinear least-squares optimization of the beam wavelength, 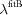, and the sample-to-detector distance, 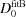. Only data from two diffraction rings at different detector positions are required to reasonably solve for 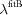 and 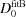; however, use of all available data across all rings from all detector positions will improve the accuracy of the fitted parameters. Once 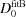 has been found, the sample-to-detector distances for all detector positions can easily be calculated from 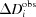.

The observables used when solving equation (17) are the previously determined values for the semi-major axes, 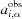, and the ratios 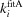, obtained from solving equation (14). As already stated, the *d* spacings, 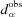, used in equation (18) are known to a high level of accuracy as a diffraction standard is used for detector calibration. The wavelength and the sample-to-detector distance are often approximately known, and these can be used as initial values to improve the speed of this nonlinear optimization. In this case the wavelength is approximately set to be at the *K* edge of Pb. The sample-to-detector distance at the detector’s furthest position for the ‘orthogonal’ case has been measured, using a tape measure, to be approximately 1977 mm.

For each detector position *i*, once the sample-to-detector distance has been calculated, the detector tilt, 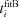, can be determined using equation (15), and the azimuthal direction of the tilt, 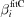, can be determined from the gradient, 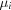, of the line of best fit through the fitted ellipse centres (Hart & Drakopoulos, 2013 ▶).

**Figure 5:**
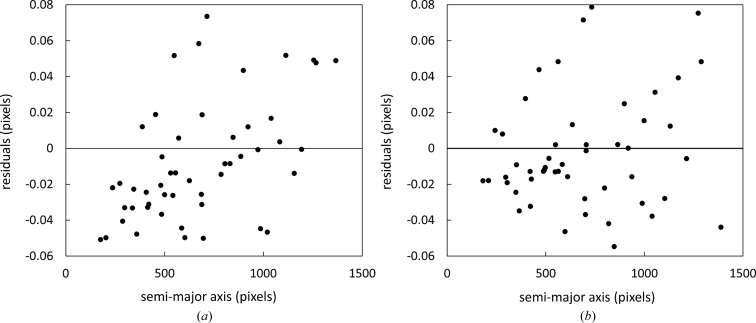
The two plots show the residuals for the semi-major axes when using equation (17) to calculate the beam energy and sample-to-detector distance. The residuals are roughly of the same size for the ‘orthogonal’ (*a*) and ‘tilted’ (*b*) cases.

The complete characterization of the detector geometry can be used to calculate theoretical diffraction rings for our 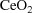 standard. These theoretical rings can then be compared with the observed rings used during calibration (Table 3 ▶). The difference between the values for the observed, 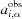, and theoretical semi-major axis lengths, 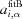, are shown in Fig. 5 ▶. The small values of these residuals together with a lack of any apparent trend give an indication of quality for both the model used in this article and the performance of the nonlinear least-squares optimization. We cannot show residuals for the beam centre as the direct X-ray beam is blocked from reaching the detector during data collection.

In practice, accurate calibration values can be attained from one set of diffraction data. Table 4 ▶ demonstrates the repeatability of our method and shows average fitted values for the beamline energy and the calibration parameters of the detector at its furthest position from the sample. Mean values are calculated from 29 separate sets of data, whilst the calculated errors are taken as the standard deviation. Also shown is the mean coefficient of determination, 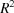, for the ellipse semi-major axes, associated with the final fit that determines the beam energy and the sample-to-detector distance. Note that the error reported for the measured beam energy reflects our uncertainty in configuring the monochromator to output an energy corresponding to the *K* edge of lead. This error is based upon our knowledge of the monochromator and a Monte Carlo method (Lenz & Ayres, 1992 ▶) which is used to estimate the error in fitting a Gaussian to the first derivative of the absorption edge.

Given accurate wavelength and detector parameters for a fixed experimental detector position (*i.e.* the subscript *i* can now be dropped), 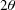 or *Q* values can be assigned to each pixel of the detector by using equations (19) (Hammersley *et al.*, 1996 ▶) and (20).
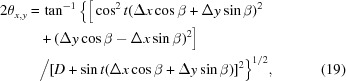
where 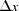 and 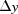 are the *x* and *y* coordinates of the pixels relative to the beam centre and are given by 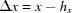 and 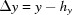.
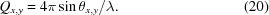



**Figure 6:**
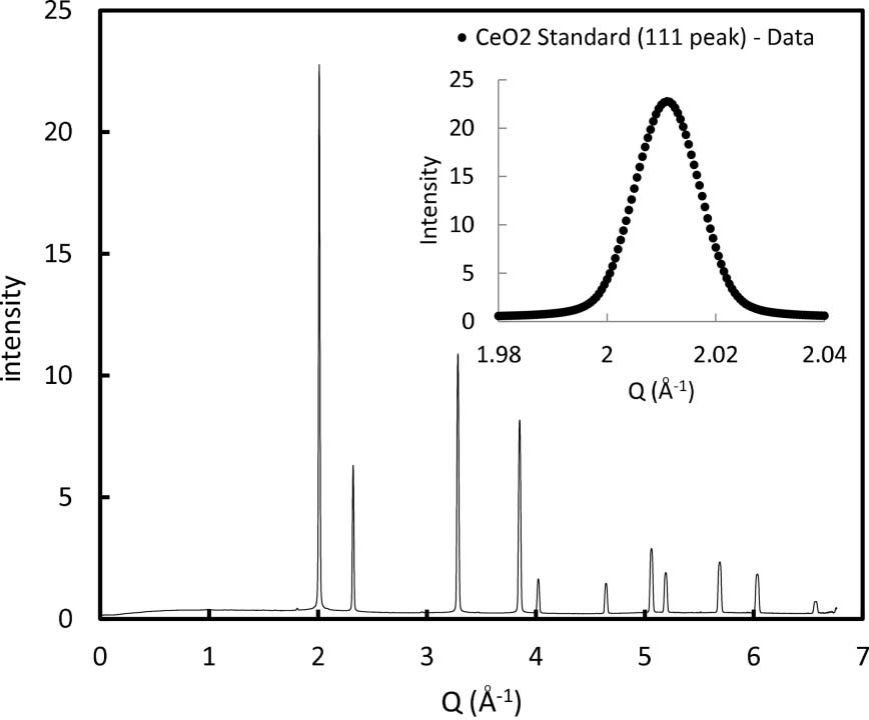
A one-dimensional diffraction pattern of 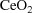 generated from a single two-dimensional diffraction image. The inset shows a single diffraction peak close up.

Now each detector pixel has associated with it not just an intensity value but also a 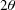 or a *Q* value. Each pixel can then be assigned to an appropriate bin of a given interval in 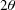 or *Q*, and a one-dimensional diffraction pattern can be directly formed (Fig. 6 ▶).

## 5. Discussion

Two-dimensional X-ray diffraction is an established scientific technique. Owing to the relationship between beam energy and sample-to-detector distance, other calibration techniques have required the accurate determination of one of these values during the course of an experiment prior to data reduction or a process of iteration. For two-dimensional detectors, accurate independent measurement of the sample-to-detector distance is difficult because the sensor plane is normally within the detector behind a protective layer. Instead the beam’s energy can be set to match the absorption *K* edge of an element such as those provided by the National Institute of Standards and Technology. This procedure can, however, be both time consuming and of limited accuracy. The measured width of the *K* edge is often appreciably wider than the theoretical width of the absorption *K* edge, as a result of several instrumental broadening effects. In addition, such an approach restricts an experiment to be performed at an energy matching the *K* edge of an available element.

With the aid of a high-precision translation stage used to alter the position of the detector along the beam axis, this article has presented an accurate method to simultaneously determine the beam energy and spatial position of the detector relative to both the beam and the sample. The entire calibration procedure, from data collection to data processing, can be accomplished within 10 min, limited only by motor speed, exposure time and computation power. The method does not require prior knowledge of any calibration parameter, other than an estimate of the sample-to-detector distance. Furthermore, each best fit of the calibration parameters is set up such that at most only two parameters are determined at a time; this increases both the accuracy and the speed at which a final solution can be found.

To help implement our method of calibration, the geometry of a cone and an intersecting plane was revisited. We have found that the point of intersection between the cone axis and its elliptical conic section has been neglected in the literature and thus have not been able to find any nomenclature for this point. It is the only common point for a set of concentric elliptical conic sections generated from a single intersecting plane. In addition, it appears that the diffraction community considers this point of intersection to be one of the focal points of the ellipse formed by the intersecting cone and plane (Norby, 1997 ▶; Hinrichsen *et al.*, 2008 ▶), in other words, that the beam centre as recorded on a detector coincides with the focal point of the surrounding diffraction ellipses. In our opinion this assumption is incorrect. By visualizing the construct of Dandelin spheres (De Villiers, 2007 ▶; Dandelin, 1822 ▶) it is seen that the cone axis does not intersect the plane at a focal point of the ellipse. Our subsequent derivation for the focal point, 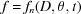 [equation (9)], and the distance from an ellipse centre to this point of intersection, 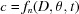 [equation (4)], supports this view.

As all diffraction ellipses recorded on a two-dimensional detector result from a single incident X-ray beam striking a sample, all diffraction ellipses share a common beam centre. The distance from the ellipse centre to the point on an ellipse that is coincident with the beam centre, 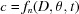, is a function of θ. This implies that the ellipse centres for the diffraction ellipses do not occupy the same point in space. The same can be said for the focal points, because in general 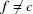. It is also the case that diffraction ellipses recorded on a single image do not have the same eccentricity: they are dissimilar in shape. The eccentricity of an ellipse is a function of θ [equation (11)].

Our new understanding of the geometry allowed us to create a method for determining the beam centre in relation to the diffraction rings recorded on a detector [equation (14)]. Our method utilizes information from multiple diffraction ellipses but does not require accurate prior knowledge of the sample-to-detector distance. To guarantee a high-quality fit it is important to ensure that the diffraction data are of high resolution and that a decent method for the determination of ellipse parameters is used. Azimuthal peak broadening effects should also be minimized by using a radially symmetric calibration sample.

Having found the beam centre for each diffraction image, which is later used in assigning a *Q* value to each detector pixel, it is actually the ratio of the sample-to-detector distance to the sine of the detector tilt [equation (15)] that is used in a subsequent calculation to determine the sample-to-detector distance itself and the beam wavelength [equation (17)].

The discrepancy between the calculated beam energy values and the theoretical value (Table 4 ▶) is considered to be within the limits of uncertainty to which the monochromator can be set to the *K* edge of Pb. We discuss errors and potential systematic corrections in Appendix *A* [App appa].

The sample-to-detector distances for the two cases differ, but this is expected as the vertical rotation axis of the stage on which our detector lies does not pass through the plane of the detecting surface.

In the ‘orthogonal’ case it was found that a small detector tilt exists. This is expected and results from the difficulties in ensuring true orthogonality of the detector to the incoming X-ray beam during experimental setup. This small tilt can explain the relatively large error in the reported value for the azimuthal direction of the tilt. The diffraction rings are very close to circular. In the ‘tilted’ case the tilt is relatively large, allowing the azimuthal tilt direction to be more easily determined. In both cases the coefficient of determination, 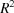, for the ellipse semi-major axes, as determined when solving equation (17), is very close to unity. Such high values of 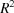 provide confidence in the quality of both the model used and the data provided.

With the help of *Nika* (Ilavsky, 2012 ▶), a software package that can calibrate and reduce two-dimensional diffraction data, we can check whether our calculated calibration parameters are self-consistent. For accurate calibration, *Nika* requires knowledge of either the beam energy or the sample-to-detector distance, but by using our calculated beam energy, consistency between the other calibrated parameters and the beam energy can be confirmed. Using an image from the ‘tilted’ case with a calculated energy of 88.0452 keV as an input (note that this is different from the reported average energy shown in Table 4 ▶, as this comparison is performed using a single set of calibration data) provides a very good agreement between our calibration parameters and those calculated using the *Nika* software package. The differences in value are sample-to-detector distance 0.055 mm, beam centre 0.009 mm and detector tilt 0.025°. Reasonable agreement was also found when using the *Fit2D* package (Hammersley *et al.*, 1996 ▶). To perform the comparison in *Fit2D* we first used the ‘tilt’ command to find the detector tilt and the beam centre, and then used the ‘calibrant’ command to find the sample-to-detector distance. For *Fit2D*, the differences in values are sample-to-detector distance 0.23 mm, beam centre 0.11 mm and detector tilt 0.052°.

Within a single procedure we have demonstrated that it is possible to accurately determine not just the beam energy but also the spatial position and orientation of the two-dimensional detector relative to the sample and the beam, and thus the complete diffraction geometry.

The performance of our approach in assigning 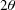 or *Q* values to each detector pixel can be further examined by looking at the peak locations for 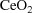 as measured from a one-dimensional diffraction pattern. Each peak position is found by locating the maxima of a fitted parabola. Table 3 ▶ compares theoretical values (generated using a lattice parameter of 5.41165 Å) for a selection of 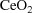 diffraction peaks with values generated from a single set of data from the ‘tilted’ case at the furthest detector position. The coefficient of determination, 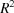, for the peak locations is 1.00–1.07 × 10^−9^. Across 29 data sets at the furthest detector position using ten diffraction rings, the average absolute percentage error in calculated peak position was 0.00238%, the standard deviation of the absolute error was 0.00186% and the maximum absolute error was 0.0124%. In other words, the majority of peak positions were calculated to be within 1 in 25 000 of the theoretical value, whilst in the worst case an error of 1 in 8000 occurred.

## 6. Conclusion

Our method of calibrating a two-dimensional detector for use in X-ray diffraction has a number of benefits. Provided multiple rings can be recorded from various positions of the detector along the beam path, the experimenter is free to choose whatever energy they desire. This energy is easily determined as part of the calibration procedure. There is no longer a requirement to know either the sample-to-detector distance or the beam wavelength, prior to calibration, as is the case with other calibration procedures such as *Fit2D* (Hammersley *et al.*, 1996 ▶) or *Nika* (Ilavsky, 2012 ▶). By collecting data from a range of detector positions and accounting for arbitrary detector tilt, we have provided a method for accurately determining all calibration parameters.

This calibration procedure is based upon our geometrical description for the intersection of a cone’s axis and its elliptical conic section.

## Figures and Tables

**Table 1:** Summary of formulae describing the ellipse formed at the intersection of a cone and a plane The cone has a half-angle of 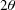. The plane is separated from the cone apex by a distance of *D* and is tilted at an angle of 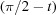 to the cone axis.

Function	Expression	
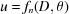	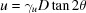 ,	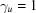
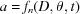	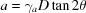 ,	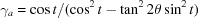
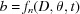	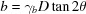 ,	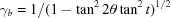
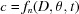	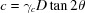 ,	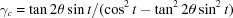
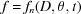	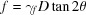 ,	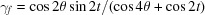
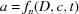	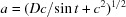	
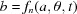	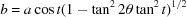	
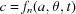	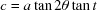	
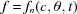	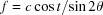	
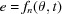	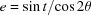	

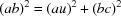		

**Table:** The subscript *i* denotes the position of the detector along the translation stage, whilst the special case of 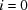 denotes the position at which the detector is initially calibrated. Calibration parameters can be found for all *i*. The subscript α denotes the diffraction ring.Parameters to be determined for detector calibration at position 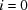.

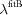	Wavelength of the X-ray beam used during calibration.
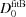	Sample-to-detector distance.
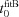	Angular tilt of the detector.
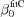	Direction of the detector tilt.
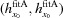	Position on the detector of the X-ray beam intersection.

**Table:** Parameters determined as part of the calibration procedure.

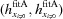	Position on the detector of the X-ray beam intersection, as the detector is translated along the beam path.
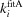	Ratio of the sample-to-detector distance to the sine of the tilt, as the detector is translated along the beam path.

**Table:** Observables required to perform the calibration procedure.

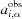	Magnitude of the semi-major axis for each diffraction ring/ellipse, as the detector is translated along the beam path.
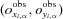	Centre of each diffraction ring/ellipse, as the detector is translated along the beam path.
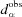	Lattice interplanar separation that results in each diffraction ring/ellipse. Mathematically this is an observable; however, in practice these values are calculated from a calibrated unit-cell parameter.
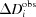	Change in distance from the calibration position, as the detector is translated along the beam path.

**Table 3:** Comparison between the calculated and theoretical values for a number of peak locations from a 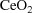 standard

{*hkl*}	‘Tilted’ case *Q* (Å^−1^)	Theoretical *Q* (Å^−1^)	Fractional error
111	2.011022	2.010994	1.41 × 10^−5^
002	2.322116	2.322096	0.86 × 10^−5^
022	3.283983	3.283940	1.33 × 10^−5^
311	3.850707	3.850761	−1.38 × 10^−5^
222	4.021878	4.021988	−2.75 × 10^−5^
004	4.644089	4.644192	−2.21 × 10^−5^
331	5.060693	5.060891	−3.91 × 10^−5^
042	5.192384	5.192364	0.38 × 10^−5^
422	5.687668	5.687950	−4.96 × 10^−5^
333/511	6.032938	6.032982	−0.74 × 10^−5^

**Table:** The 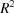 coefficient for the semi-major axis is also shown for the final fit that determines the X-ray beam energy and the sample-to-detector distance.

	‘Orthogonal’ calculated	‘Orthogonal’ measured
Beam energy, 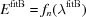	88.0399 (7) keV	88.012 (42) keV (Pb *K* edge)
Sample-to-detector distance, 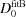	1976.497 (10) mm	1977 (1) mm
Detector tilt, 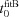	0.096 (12)°	0.0 (5)°
Azimuthal tilt direction, 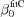	9.8 (95)°	None
Beam centre, *x* coordinate, 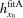	1398.673 (5) pixels	
Beam centre, *y* coordinate, 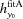	1445.558 (11) pixels	
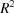 coefficient	1.00–1.45 (25) × 10^−8^	

**Table:** 

	‘Tilted’ calculated	‘Tilted’ measured
Beam energy, 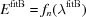	88.0442 (27) keV	88.012 (42) keV (Pb *K* edge)
Sample-to-detector distance, 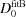	1991.805 (24) mm	1992 (1) mm
Detector tilt, 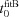	6.375 (3)°	6.6 (5)°
Azimuthal tilt direction, 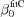	0.234 (24)°	0.0 (5)°
Beam centre, *x* coordinate, 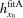	1442.381 (3) pixels	
Beam centre, *y* coordinate, 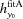	1445.624 (3) pixels	
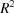 coefficient	1.00–1.45 (75) × 10^−8^	

## Footnotes

1 For the purpose of this article a diffraction ring can be circular or elliptical. A diffraction ring recorded upon an orthogonal detector will be circular in shape; however, if the detector is tilted then the diffraction ring will be elliptical in shape.

## Appendix A Sources of error

There are a number of factors that can affect the accuracy of the calibration procedure, some random and some systematic. Note, however, that we are working in the superresolution paradigm where we are determining parameters at a scale that is up to two orders of magnitude better than the instrument resolution (given by the energy bandwidth of the monochromator, the sample thickness and the angular resolution of the detector).

The thermal stability of the sample and detector environment can be controlled; however, it is normal for some thermal variation to occur within an experimental hutch. This thermal variation affects not just the expansion of the calibration standard but also the displacement of the stage upon which the detector rests. The entire instrumental setup including the steel support structures is subject to thermal expansion. The translation stage also exhibits a systematic error due to a variation in flatness of the floor-mounted guide rails, which thus creates a variation in tilt and displacement at the level of the detector. An additional random error can be attributed to mechanical runout.

Geometrical attenuation effects can also be considered, in particular the attenuation of the X-rays passing through the standard and the attenuation that occurs within the scintillating plane of the flat-panel detector. Both effects lead to a systematic shift in the recorded position for the centre of mass of a diffraction peak as recorded by the detector. Such attenuation affects can be quantified and applied to the calibration procedure. These corrections should improve the accuracy of the calculated beam energy; however, it could be argued that, unless equivalent transformations to correct for attenuation can be applied to experimental data for all *Q*, these corrections should not be applied to the calibration procedure. For the purpose of facilitating high-quality data reduction we feel it is better to allow the calibration parameters to take values that compensate for the attenuated diffraction geometry, assuming that the experimental data cannot be mapped into this attenuation-free ‘perfect’ space.

### A1. Error due to thermal expansion of the calibration standard

At room temperature the coefficient of linear thermal expansion for cerium dioxide is approximately 10^−5^ K^−1^. This translates to a thermal dependence in the calculated energy of approximately 0.8 eV K^−1^. The thermal stability of the experimental hutch at I12 is approximately  K. This thermal dependence can be considered as an uncertainty in the calibration procedure, or if the temperature of the standard is known at the time of data collection, then the lattice parameter of the standard can be suitably scaled.

### A2. Error due to detector stage displacement

The detector stage displacement is an important parameter for the calibration procedure, as the diffraction patterns recorded at the various detector positions define the geometry of the diffraction cones. By using a Renishaw XL-80 interferometer we are able to determine the displacement of the translation stage more accurately. Compared to the nominal displacements used in the main body of this article, we found a systematic error in displacement of up to 125 µm in size dependent upon translation stage position. By performing repeated movements of the translation stage we were able to determine that runout and thermal expansion of the translation assembly due to the motor contribute to an uncertainty in the stage position of up to  µm, which results in a  eV uncertainty in the calculated energy. Correcting for the nominal sample-to-detector distances in a representative calibration calculation reduced the spread in residuals for the ellipse semi-major axis [see equation (17) and Fig. 5 ▶] by approximately 30%. However, by applying this correction for the pseudo-random detector position, underlying drifts in the residuals (ordered by increasing semi-major axis length) grouped by diffraction image become apparent.

These drifts can be removed by considering X-ray attenuation of the sample and the detector’s scintillating surface, as presented in the next section.

### A3. Error due to X-ray attenuation

X-ray attenuation at the sample and the detector’s scintillator shift the effective position of a diffraction ring. In the case of sample attenuation (in a planar arrangement), an integral over all possible diffraction paths results in a shift in the centre of mass of the diffraction peak towards lower *Q*. A similar, but smaller, centre of mass shift occurs in the scintillating volume, due to the absorption of photons arriving at an angle to the detector surface. For an orthogonal detector with scintillator thickness , the corresponding change in spatial peak position, , at Bragg angle θ can be approximated by

where

and  is the linear attenuation coefficient of the scintillator.

For an orthogonal detector and a planar sample of thickness , the change in peak position due to attenuation at the sample, , at Bragg angle θ can be approximated by

where

and  is the linear attenuation coefficient of the sample.

At 88 keV correcting for attenuation at the sample ( cm^−1^) and the detector ( cm^−1^) reduces the calculated beam energy by 1.3 eV. Note that the effect of the increasing shift in diffraction peak location towards lower *Q*, with increasing diffraction angle, can be considered equivalent to diffraction from a point source onto a curved virtual detector surface/scintillator of infinitesimal thickness.

### A4. Other sources of error

This list of errors is by no means exhaustive but is representative of the types of corrections that can be implemented to improve the calibration procedure. Other sources of error include the flatness of the scintillator surface, the detection efficiency of the pixels, X-ray attenuation at the scintillator for a tilted detector and energy drift of the monochromator. Errors can be segregated in terms of whether they are directly associated with our technique or with the process of attaining calibration data.

In our measurements we experienced experimental errors of 3 eV, which we believe can be attributed to monochromator instability. The monochromator used does not have active thermal stabilization and the ‘orthogonal’ and ‘tilted’ data sets were collected two hours apart. We believe that energy drift from the monochromator can account for the majority of the energy difference. The monochromator was configured to use the Si 111 reflection. Using Bragg’s law and  , with  eV and  keV, gives   µrad.
